# Excitatory Neuronal Responses of Ca^2+^ Transients in Interstitial Cells of Cajal in the Small Intestine

**DOI:** 10.1523/ENEURO.0080-18.2018

**Published:** 2018-04-06

**Authors:** Salah A. Baker, Bernard T. Drumm, Karolina E. Skowronek, Benjamin E. Rembetski, Lauren E. Peri, Grant W. Hennig, Brian A. Perrino, Kenton M. Sanders

**Affiliations:** 1 Department of Physiology and Cell Biology, University of Nevada School of Medicine, Reno, NV 89557; 2 Department of Pharmacology, University of Vermont, Burlington, VT 05405

**Keywords:** c-Kit, enteric neurotransmission, gastrointestinal motility

## Abstract

Interstitial cells of Cajal (ICC) regulate smooth muscle excitability and motility in the gastrointestinal (GI) tract. ICC in the deep muscular plexus (ICC-DMP) of the small intestine are aligned closely with varicosities of enteric motor neurons and thought to transduce neural responses. ICC-DMP generate Ca^2+^ transients that activate Ca^2+^ activated Cl^-^ channels and generate electrophysiological responses. We tested the hypothesis that excitatory neurotransmitters regulate Ca^2+^ transients in ICC-DMP as a means of regulating intestinal muscles. High-resolution confocal microscopy was used to image Ca^2+^ transients in ICC-DMP within murine small intestinal muscles with cell-specific expression of GCaMP3. Intrinsic nerves were stimulated by electrical field stimulation (EFS). ICC-DMP exhibited ongoing Ca^2+^ transients before stimuli were applied. EFS caused initial suppression of Ca^2+^ transients, followed by escape during sustained stimulation, and large increases in Ca^2+^ transients after cessation of stimulation. Basal Ca^2+^ activity and the excitatory phases of Ca^2+^ responses to EFS were inhibited by atropine and neurokinin 1 receptor (NK1) antagonists, but not by NK2 receptor antagonists. Exogenous ACh and substance P (SP) increased Ca^2+^ transients, atropine and NK1 antagonists decreased Ca^2+^ transients. Neurokinins appear to be released spontaneously (tonic excitation) in small intestinal muscles and are the dominant excitatory neurotransmitters. Subcellular regulation of Ca^2+^ release events in ICC-DMP may be a means by which excitatory neurotransmission organizes intestinal motility patterns.

## Significance Statement

Interstitial cells of Cajal (ICC) are innervated by enteric motor neurons and thought to transduce neural responses in GI muscles. Ca^2+^ transients, due to Ca^2+^ release from Ca^2+^ intracellular stores, mediate electrophysiological events in ICC by activation of Ca^2+^-activated Cl^-^ channels (CaCCs). Neural responses in ICC in the deep muscular plexus (ICC-DMP) of the small intestine were studied by confocal imaging of Ca^2+^ transients in these cells. Excitatory neural input was due to cholinergic and peptidergic neurotransmitters [acetylcholine (ACh) and neurokinins], as excitatory effects on Ca^2+^ transients were blocked by atropine and neurokinin receptor antagonists. Neurokinins are the dominant excitatory regulators of Ca^2+^ transients in ICC-DMP. ICC-DMP are innervated by enteric motor neurons and mediate significant excitatory responses in intestinal muscles.

## Introduction

Muscles of the gastrointestinal (GI) tract are innervated by both excitatory and inhibitory enteric motor neurons (Furness, 2012), and motility patterns of the gut depend on the outputs of the enteric nervous system. Neural inputs are overlaid on the basal excitability of the smooth muscle cells (SMCs) that line the walls of GI organs. SMC excitability is determined by ionic conductances and Ca^2+^ sensitization mechanisms intrinsic to these cells but also by interstitial cells that are electrically coupled to SMCs. Together SMCs and interstitial cells, i.e., interstitial cells of Cajal (ICC) and platelet-derived growth factor receptor α-immunopositive (PDGFRα^+^) cells (Komuro, 2006; Sanders and Ward, 2006; Iino et al., 2009; Blair et al., 2012; Baker et al., 2013), form an electrical syncytium, known as the SIP syncytium (Sanders et al., 2012). It is the integrated output of these cells that determines the basal excitability of GI smooth muscle tissues and ultimately the responses to enteric motor neurons and other higher order regulatory pathways (Sanders et al., 2014a).

In the small intestine, a network of ICC in the myenteric region (ICC-MY) serves as the pacemaker cells that generate and actively propagate electrical slow waves and organize contractile activity into a phasic pattern that underlies segmental contractions (Langton et al., 1989; Ward et al., 1994; Ordög et al., 1999; Sanders et al., 2014a). Another class of ICC are distributed within smooth muscle bundles in the deep muscular plexus (ICC-DMP; Rumessen et al., 1992; Zhou and Komuro, 1992) throughout the smooth muscle organs of the GI tract. ICC-DMP are innervated by motor neurons and transduce part of the input from enteric motor neurons (Wang et al., 2003b; Iino et al., 2004; Ward et al., 2006). This conclusion is based on the fact that ICC-DMP are: (1) closely apposed to varicosities of enteric motor neurons, forming synaptic-like contacts, i.e., <20 nM (Rumessen et al., 1992; Zhou and Komuro, 1992); (2) express major receptors for enteric motor neurotransmitters (Sternini et al., 1995; Vannucchi et al., 1997; Chen et al., 2007); (3) display evidence of receptor binding, receptor internalization, and translocation of signaling molecules on nerve stimulation (Wang et al., 2003b; Iino et al., 2004); and (4) electrically coupled to SMCs via gap junctions (Daniel et al., 1998; Daniel and Wang, 1999; Seki and Komuro, 2001). Experiments in other regions of the GI tract, where ICC-IM are lost in mutant animals have shown distinct changes in motor neurotransmission in the absence of ICC (Daniel and Posey-Daniel, 1984; Burns et al., 1996; Komuro et al., 1999; Ward et al., 2000; Klein et al., 2013). Nevertheless, there is controversy about the importance of ICC in neurotransmission, and some investigators have argued that ICC are not important elements in enteric nerve responses (Goyal and Chaudhury, 2010; Goyal, 2016).

A fundamental mechanism involved in the activation of ICC (as pacemakers and in regulating the excitability of GI muscles) is Ca^2+^ release from intracellular stores (van Helden and Imtiaz, 2003; Lee et al., 2007; Baker et al., 2016; Drumm et al., 2017). Ca^2+^ release is important because it activates Ca^2+^-activated Cl^-^ channels (CaCCs), encoded by *Ano1*, that are strongly expressed in ICC (Chen et al., 2007; Gomez-Pinilla et al., 2009; Zhu et al., 2015). We have used mice expressing Ca^2+^ sensors specifically in ICC to investigate the Ca^2+^ transients generated by ICC in intact intestinal muscles (Baker et al., 2016; Drumm et al., 2017).

Excitatory neurotransmission in the gut is mediated predominantly via cholinergic neurotransmitters and neurokinins. The tachykinin (TKs) family of peptides [substance P (SP), neurokinin A (NKA) and NKB] is expressed throughout the GI tract (Holzer and Holzer-Petsche, 2001; Cipriani et al., 2011; Mitsui, 2011; Steinhoff et al., 2014). SP, NKA, and NKB are preferentially mediated through the stimulation of neurokinin 1 receptor (NK1), NK2, and NK3 G protein-coupled receptors. Both NK1 and NK2 receptors mediate contractile effects in the gut. Smooth muscle electrical, and motor events induced by electrical field stimulation (EFS) can involve both NK1 and NK2 receptors. But functional evidence supports the involvement of the NK1 subtype in mediating nonadrenergic noncholinergic (NANC) contractions to EFS in the mouse small intestine (Iino et al., 2004; De Schepper et al., 2005).

In the present study, we investigated the hypothesis that a major mechanism by which enteric motor neurotransmitters regulate ICC is through modulation of Ca^2+^ release events. To test this hypothesis, we explored whether excitatory neural inputs to ICC-DMP are coupled to Ca^2+^ release and characterized the nature of the Ca^2+^ responses that constitute this transduction pathway for postjunctional excitatory transmission.

## Materials and Methods

### Animals

GCaMP3-floxed mice (B6.129S-*Gt(ROSA)26Sor^tm38(CAG-GCaMP3)Hze^*/J) and their wild-type siblings (C57BL/6) were acquired from The Jackson Laboratory and crossed with Kit-Cre mice (*c-Kit^+/Cre-ERT2^*), provided by Dr. Dieter Saur (Technical University Munich, Munich, Germany). Kit-Cre-GCaMP3 mice (both sexes) were injected with tamoxifen at six to eight weeks of age (2 mg for three consecutive days), as previously described (Baker et al., 2016) to activate Cre recombinase and GCaMP3 expression. 15 days after tamoxifen injection, Kit-Cre-GCaMP3 mice were anaesthetized by isoflurane inhalation (Baxter) and killed by cervical dislocation. All animals used for these experiments were handled in compliance with the National Institutes of Health Guide for the Care and Use of Laboratory Animals, and the protocols were approved by the Institutional Animal Use and Care Committee at the University of Nevada, Reno.

### Tissue preparation

Segments of jejunum (2 cm in length) were removed from mice and bathed in Krebs-Ringer bicarbonate solution (KRB). Jejunal segments were opened along the mesenteric border and luminal contents were washed away with KRB. The mucosa and sub-mucosa layers were removed by sharp dissection, and the remaining tunica muscularis was pinned flat within a Sylgard coated dish.

### Drugs and solutions

Tissues were maintained and perfused with KRB containing 120.35 mmol/l NaCl, 5.9 mmol/l KCl, 15.5 mmol/l NaHCO_3_, 1.2 mmol/l NaH_2_PO_4_, 1.2 mmol/l MgCl_2_, 2.5 mmol/l CaCl_2_, and 11.5 mmol/l glucose. The KRB was bubbled with a mixture of 97% O_2_-3% CO_2_ and warmed to 37 ± 0.2°C.

All drugs were purchased from Tocris Bioscience and dissolved in the solvents recommended by the manufacturer to obtain stock solutions. Final concentrations used in experiments were obtained by dilution into KRB.

### Fluorescence-activated cell sorting (FACS), RNA extraction, and quantitative PCR (qPCR)

Jejunal ICC were dispersed from *Kit^+/copGFP^* mice as previously described (Zhu et al., 2009; Zhu et al., 2011). ICC were sorted and purified by FACS (FACSAria II; Becton-Dickinson) using an excitation laser (488 nm) and emission filter (530/30 nm). Sorting was performed using a 130-μm nozzle and a sheath pressure of 12 psi.

RNA was prepared from sorted ICC and dispersed jejunal cells of the tunica muscularis before sorting using an illustra RNAspin Mini RNA Isolation kit (GE Healthcare). The PCR primers used and their GenBank accession numbers are provided in Table 1. qPCR was performed using SYBR green chemistry on the 7500 HT Real-time PCR System (Applied Biosystems) and analyzed, as previously described (Baker et al., 2016). All datasets were normalized to the housekeeping gene *Gapdh*.

**Table 1.** Summary table of cholinergic and neurokinin receptor primer sequences

Gene	Sequence	GenBank Accession Number
mGapdh-F	AGACGGCCGCATCTTCTT	NM_008084
mGapdh-R	TTCACACCGACCTTCACCAT	
mChrm2-F	GGTGTCTCCCAGTCTAGTGCAAGG	NM_203491
mChrm2-R	ATGTCTGCCTAGAGTTGTCATCTTTGGA	
mChrm3-F	TGTGGCCAGCAATGCTTCTGTCATGA	NM_033269
mChrm3-R	CCACAGGACAAAGGAGATGACCCAAG	
mTacr1-F	GTGGTGAACTTCACCTACGCAGTC	NM_009313
mTacr1-R	GCCATGTATGCTTCAAAGGCCACAG	
mTacr2-F	CCATCGCCGCTGACAGGTACA	NM_009314
mTacr2-R	GGCCCCCTGGTCCACAGTGA	

Table lists muscarinic (M2, M3) and neurokinin (NK1, NK2) receptor gene transcripts that were measured in this study including their name, primer sequences, and gene bank accession numbers.

### Calcium imaging

Jejunal muscle sheets (5.0 × 10.0 mm) were pinned to the base of a 5-ml, 60-mm diameter Sylgard-coated dish. The muscles were perfused with warmed KRB solution at 37°C for an equilibration period of 1 h. Fluorescence imaging was performed with a spinning-disk confocal microscope (CSU-W1 spinning disk; Yokogawa Electric Corporation) mounted to an upright Nikon Eclipse FN1 microscope equipped with a 60× 1.0 NA CFI Fluor lens (Nikon Instruments Inc). GCaMP3, expressed solely in ICC, was excited at 488 nm using a laser coupled to a Borealis system (ANDOR Technology). Emitted fluorescence (>515 nm) was captured using a high-speed EMCCD Camera (Andor iXon Ultra; ANDOR Technology). Image sequences were collected at 33 fps using MetaMorph software (Molecular Devices Inc). Additional Ca^2+^ imaging data were acquired with an Eclipse E600FN microscope (Nikon Inc.) equipped with a 60× 1.0 CFI Fluor lens (Nikon instruments Inc). In this system, GCaMP3 was excited at 488 nm (T.I.L.L. Polychrome IV), as previously described (Baker et al., 2013). All Ca^2+^ imaging experiments were performed in the presence of nicardipine (100 nM) to minimize contractile movements.

### Calcium event analysis

Analysis of Ca^2+^ activity in ICC-DMP was performed, as described previously (Baker et al., 2016). Briefly, movies of Ca^2+^ activity in ICC-DMP were converted to a stack of TIFF (tagged image file format) images and imported into custom software (Volumetry G8c, GW Hennig) for analysis. Tissue movement was stabilized to ensure accurate measurement of Ca^2+^ transients from ICC-DMP. Whole cell ROIs were used to generate spatio-temporal (ST) maps of Ca^2+^ activity in individual ICC-DMP. ST maps were then imported as TIFF files into ImageJ (version 1.40, National Institutes of Health; http://rsbweb.nih.gov/ij) for *post hoc* quantification analysis of Ca^2+^ events.

### Experimental design and statistical analysis

Ca^2+^ event frequency in ICC-DMP was expressed as the number of events fired per cell per second (s^−1^). Ca^2+^ event amplitude was expressed as ΔF/F_0_, the duration of Ca^2+^ events was expressed as full duration at half maximum amplitude (FDHM), and Ca^2+^ event spatial spread was expressed as μm of cell propagated per Ca^2+^ event. Unless otherwise stated, data are represented as mean ± SEM. Statistical analysis was performed using either a Student’s *t* test or with an ANOVA with a Dunnett *post hoc* test where appropriate. In all statistical analyses, *p* < 0.05 was taken as significant; *p* < 0.05 are represented by a single asterisk (*), *p* < 0.01 are represented by two asterisks (**), and *p* < 0.001 are represented by three asterisks (***). When describing data throughout the text, n refers to the number of animals used in that dataset while c refers to the numbers of cells used in that same dataset.

## Results

### Postjunctional modulation of Ca^2+^ signaling in ICC-DMP by enteric nerve stimulation

**Figure 1.**
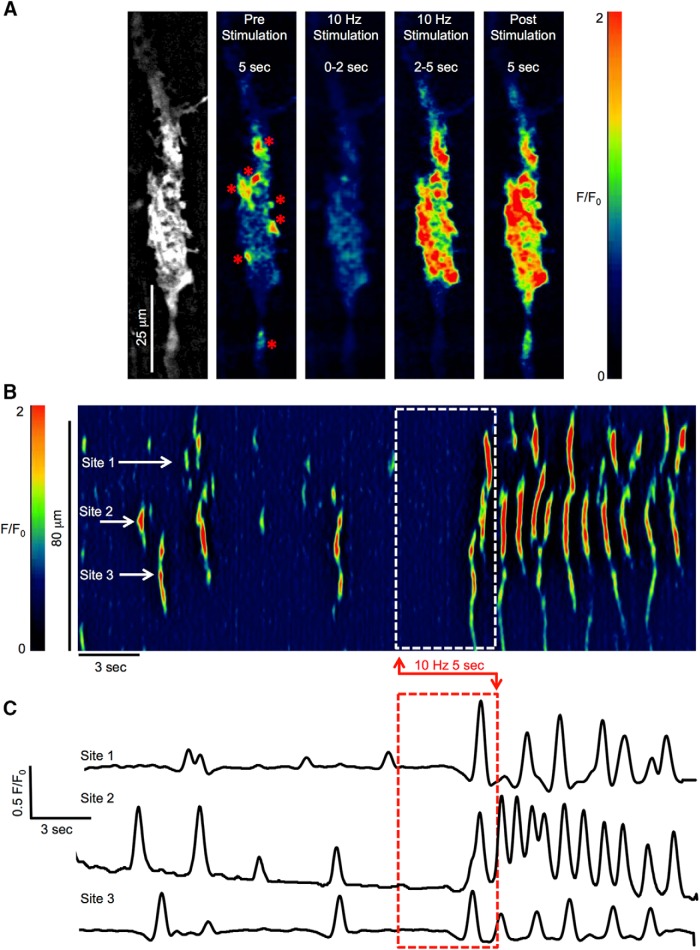
ICC-DMP Ca^2+^ transient responses to nerve stimulation. ***A***, Time-lapse montage showing postjunctional Ca^2+^ responses to EFS (10 Hz; 0.5-ms duration; 5 s) on an ICC-DMP *in situ*. An image of the GCaMP3 signal in the cell is shown in the leftmost panel. Scale bar for all panels: 25 μm. A color-coded overlay and calibration scale was imported to depict fluorescence intensity (*F*/*F*_0_) and enhance visualization of Ca^2+^ sites. Low fluorescence areas are indicated in dark blue or black. High-intensity fluorescence areas are indicated in red and orange. The “pre stimulation” panel shows a summed image of Ca^2+^ activity within the cell for 5 s before the onset of EFS, Ca^2+^ firing sites are marked with red asterisks. Panels showing the summed Ca^2+^ activity for the initial 2 s of EFS, the final 3 s of EFS and 5-s post-EFS are also shown. ***B***, Representative ST map of Ca^2+^ transients in ICC-DMP shown in ***A***. EFS duration is indicated by the dashed white box. The firing activities of three sites highlighted on the ST map are plotted in ***C***, and the timing of EFS is indicated by the dashed red box.

ICC-DMP displayed intracellular Ca^2+^ transients that fired in a stochastic manner (Fig. 1), as reported previously (Baker et al., 2016). Ca^2+^ transients were generated at multiple sites along the length of individual ICC-DMP and were typically localized, demonstrating no mechanism for active or regenerative propagation of these events within individual cells or between cells and no extrinsic mechanism of entrainment, as has been previously suggested (Huizinga et al., 2014). Ca^2+^ transients in ICC-DMP exhibit a range of frequencies, amplitudes, durations and spatial spread (Baker et al., 2016). ICC are thought to be intermediaries in enteric neurotransmission, relaying signals from enteric neurons to smooth muscle cells, that are electrically coupled to ICC (Daniel et al., 1998; Daniel and Wang, 1999). Therefore, we investigated how Ca^2+^ transients are modulated by enteric neurons activated by EFS.

EFS (10 Hz, 0.5 ms for 5-s trains) caused two distinct Ca^2+^ responses: (1) an initial inhibitory phase; (2) an excitatory response that occurred largely after cessation of EFS (Movie 1). The initial inhibitory response at the onset of EFS lasted about ∼2 s. During this phase, Ca^2+^ transients in ICC-DMP ceased (Fig. 1*A–C*). In the final 3 s of EFS, Ca^2+^ transients escaped from inhibition leading to an excitatory response that persisted into the period after cessation of the stimulus (Fig. 1*A*). These effects are illustrated by an ST map and Ca^2+^ activity traces in Figure 1*B*,*C*. This example demonstrates that in the final 3 s of EFS and particularly in the 5 s after cessation of EFS, Ca^2+^ transients were increased relative to the control period, and firing sites within ICC-DMP increased their firing frequency. We also found that the initiation sites for Ca^2+^ transients varied temporally in response to EFS (Fig. 1*B*). These responses were mediated by neuronal inputs, as they were blocked by tetrodotoxin (TTX, 1 μM, data not shown). As above, after the onset of EFS, an inhibitory response phase occurred, but in subsequent experiments we concentrated on the excitatory aspects of the neural responses.

Movie 1. ICC-DMP Ca^2+^ transient responses to enteric neuronal stimulation. Movie of intracellular Ca^2+^ transients in ICC-DMP labeled with the genetically encoded Ca^2+^ indicator GCaMP3 in response to EFS (10 Hz, for 5 s; real-time playback). The top left FOV shows typical elongated ICC-DMP using a 60× objective (original recordings). Note that Ca^2+^ transients fired in stochastic fashion the blue bit-masked cell. The right window shows Ca^2+^ transient particles thresholded (SNR >= 25 dB, to facilitate visualization of active signals) after differentiation (Δt = 0.5 s) and smoothing (Gaussian 1.0 SD, box size = 3.3 µm) as shown in the middle window. Scale bar in top left window is 15 μm and pertains to all windows. The blue overlay of ICC-DMP in the FOV (blue bit-masked cell) was used to construct an ST map of Ca^2+^-induced fluorescence intensity across the diameter of the cell, which better displays the firing and propagation of Ca^2+^ transients along the length of the cell in response to EFS (lower panel; EFS duration is indicated with the yellow box). The bottom panel shows active area of Ca^2+^ transients across the FOV (area of active particles). Note the caseation of Ca^2+^ transients in response to EFS and enhanced Ca^2+^ firing during post stimulus period. Scale bar in the lower ST map and bottom active area map: 50 μm. 10.1523/ENEURO.0080-18.2018.video.1

**Figure 2.**
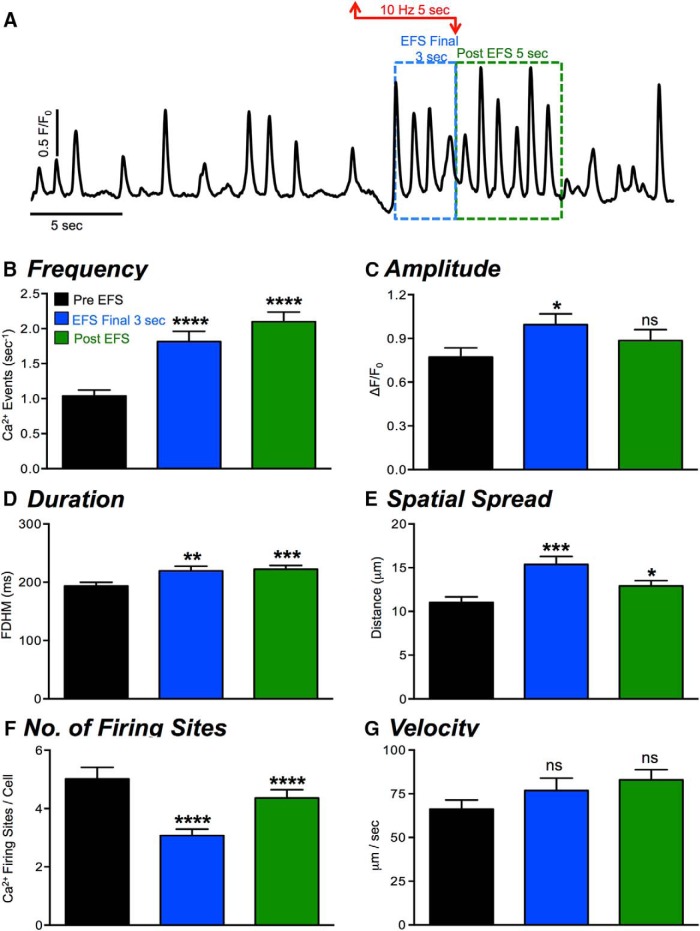
Effects of nerve stimulation (EFS) on Ca^2+^ transients in ICC-DMP. ***A***, Representative trace representing Ca^2+^ transients in ICC-DMP in response to EFS (10 Hz; 5 s). The period of EFS is indicated by the red arrowed line. Excitatory responses during the final 3 s of EFS, indicated by the dashed blue box, and during the post-EFS period (5 s), highlighted by the green box. ***B–G***, Summary data quantifying the effects of EFS on ICC-DMP: Ca^2+^ transient frequency (***E***), amplitude (***F***), duration (***G***), spatial spread (***H***), number of Ca^2+^ firing sites (***I***), and Ca^2+^ transient velocity (***J***) were analyzed and shown; *n* = 23, c = 56. All statistical analyses shown are compared to control values; ns = *p* > 0.05, *p < 0.05, **p < 0.01, ***p < 0.001, ***p < 0.0001.

The excitatory Ca^2+^ response to EFS was quantified during the final 3 s of EFS (Fig. 2*A*, blue dashed box) and in the 5 s immediately following EFS (post-EFS; Fig. 2*A*, green dashed box). In the pre-EFS period, the control frequency of Ca^2+^ transients was 1.04 ± 0.08 events s^−1^, and this was increased significantly during the final 3-s period of EFS to 1.8 ± 0.15 events s^−1^ (Fig. 2*B*, *p* < 0.0001, *n* = 23, c = 56). The frequency of Ca^2+^ transients in the post-EFS period was also significantly increased from control, firing on average at 2.1 ± 0.1 events s^−1^ (Fig. 2*B*, *p* < 0.0001, *n* = 23, c = 56). There was a significant increase in Ca^2+^ transient amplitude in the final 3 s of EFS from 0.8 ± 0.06-1.1 ± 0.05 ΔF/F_0_ (Fig. 2*C*, *p* < 0.05, *n* = 23, c = 56), although there was no significant increase in amplitude in the post-EFS period compared to control (Fig. 2*C*, *p* > 0.05, *n* = 23, c = 56). Ca^2+^ transient duration increased in the final 3 s of EFS from 193 ± 3.7 to 219.6 ± 7.9 ms (Fig. 2*D*, *p* < 0.01, *n* = 23, c = 56) and was also significantly increased in the post-EFS period, increasing to 222 ± 6.5 ms (Fig. 2*D*, *p* < 0.001, *n* = 23, c = 56). Ca^2+^ transient propagation spread was also increased in the final 3 s of EFS from 11 ± 0.6 to 15.4 ± 0.9 μm (Fig. 2*E*, *p* < 0.001, *n* = 23, c = 56) and was also significantly increased, as compared to control, in the post-EFS period, with Ca^2+^ transients propagating an average of 12.9 ± 0.6 μm (Fig. 2*E*, *p* < 0.05, *n* = 23, c = 56). The number of Ca^2+^ firing sites in ICC-DMP was decreased significantly during the final 3 s of EFS (*p* < 0.001) and during the post-EFS period (*p* < 0.001; Fig. 2*F*, *n* = 23, c = 56). This is likely a result of the increased propagation spread of Ca^2+^ transients during these periods, as shown in Figure 2*E*. As the frequency of Ca^2+^ transients increased and they propagated over longer distances, individual firing sites may summate to create the increase in propagation distances observed during the final seconds of EFS and post-EFS. This could lead to an apparent reduction in firing sites, as the underlying sites were masked by propagating Ca^2+^ waves. A small increase in Ca^2+^ transient propagation velocity, that did not reach significance, was also observed during the final 3 s of EFS and during the post-EFS period (*p* < 0.05; Fig. 2*G*, *n* = 23, c = 56).

### EFS-evoked frequency-dependent excitatory Ca^2+^ responses in ICC-DMP

**Figure 3.**
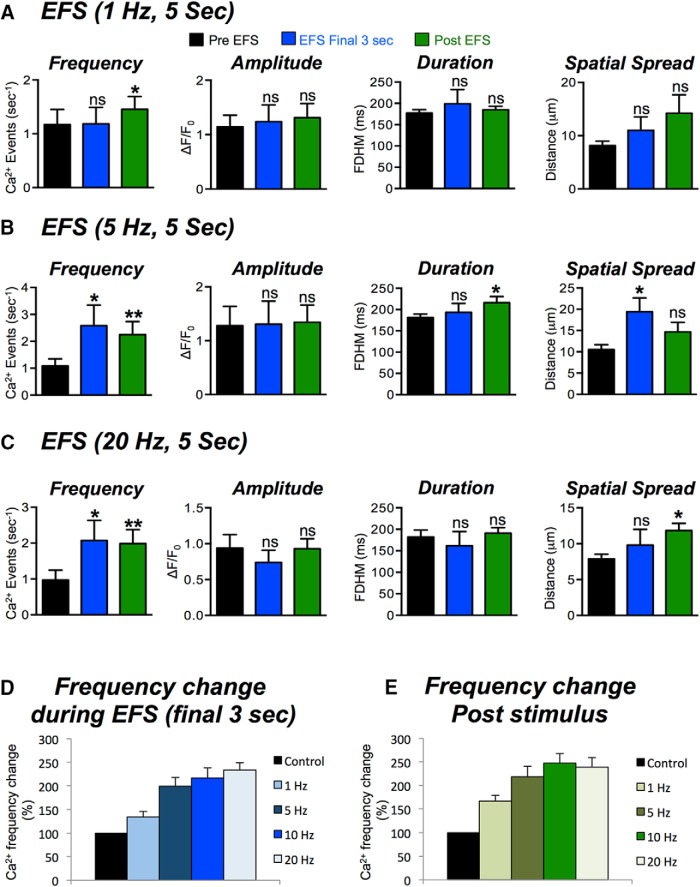
Frequency dependence of Ca^2+^ transient responses to EFS. ***A***, Summary data showing the excitatory effects of EFS (1 Hz for 5 s) on Ca^2+^ transients in ICC-DMP during the final 3 s of EFS and during the poststimulus period (5 s following termination of EFS). Ca^2+^ transient parameters shown include: frequency (s^−1^), amplitude (ΔF/F_0_), duration (FDHM), and spatial spread (μm) of Ca^2+^ transients. ***B***, Summary data showing the effects of EFS (5 Hz; 5 s) on Ca^2+^ transient parameters. ***C***, Summary data showing the effects of EFS (20 Hz; 5 s) on Ca^2+^ transient parameters. ***D***, Percentage (%) change of Ca^2+^ transient firing frequency at all frequencies of EFS tested (1–20 Hz; net percentage change normalized to control) during the final 3 s of EFS and during the poststimulus period. ***E***, Note the frequency-dependent effects of EFS on Ca^2+^ transient responses. Summary data in all panels shows the include 5 s before EFS, the final 3 s during EFS and 5-s post-EFS; ns = *p* > 0.05, *p < 0.05, **p < 0.01.

We examined whether the Ca^2+^ responses in ICC-DMP were dependent on the frequency of EFS. EFS was applied to muscles from 1 to 20 Hz (0.5 ms, 5-s trains). No changes in Ca^2+^ transient parameters were resolved during 1-Hz stimulation (Fig. 3*A*, *n* = 5, c = 16), although a significant increase in the frequency of Ca^2+^ transients occurred in the post-EFS period (Fig. 3*A*, *p* < 0.05, *n* = 5, c = 16). Higher EFS frequencies (5, 10, and 20 Hz) increased Ca^2+^ transients significantly during EFS (final 3 s) and during the post stimulus period (Fig. 3*B*,*C*). For example, 5 Hz EFS increased the firing frequency (final 3 s) to 2.5 ± 0.6 events s^−1^, which was significantly greater than control values of 1.6 ± 0.4 events s^−1^ (*p* < 0.05, *n* = 5, c = 16). EFS 5 Hz also increased Ca^2+^ transient frequency during the post-EFS period to 2.5 ± 0.4 events s^−1^, as compared to 1.6 ± 0.4 events s^−1^ in control (*p* < 0.01, *n* = 5, c = 16). During EFS, the amplitude and duration of Ca^2+^ transients were not significantly changed at all frequencies tested (*p* > 0.05). However, Ca^2+^ transient duration increased during the post-EFS period at 5 Hz from 198 ± 10 to 228 ± 13.1 ms (*p* < 0.05, *n* = 5, c = 16). The spread of Ca^2+^ transients was not significantly affected by 1-Hz EFS but increased significantly at 5 Hz during EFS (final 3 s; increased from 10.1 ± 0.9 to 18.6 ± 2.6 μm (*p* < 0.05, *n* = 5, c = 16). At 20 Hz, the spatial spread increased from 8.5 ± 0.6 to 11.9 ± 1.2 μm during EFS (*p* < 0.05, *n* = 5, c = 14). The change in firing frequency (% change) for each stimulus 1, 5 10, and 20 Hz was calculated and plotted in Figure 3*D*,*E* during EFS (final 3 s; Fig. 3*D*) and after the stimulus period (Fig. 3*E*). The firing of Ca^2+^ transients was dependent on the stimulus frequency during both periods (Fig. 3*D*,*E*).

### Expression of excitatory cholinergic and neurokinin receptors in ICC

Excitatory neurotransmitters mediate responses by binding to specific post junctional receptors. In the case of excitatory enteric neurotransmission, responses have been attributed to muscarinic (M2 and M3) receptors and neurokinin (NK1 and NK2) receptors expressed in small intestinal muscles (Lavin et al., 1998; Stadelmann et al., 1998; Iino et al., 2004; Faussone-Pellegrini and Vannucchi, 2006). In this study, we sorted ICC (CopGFP-Kit^+^ cells) from small intestinal muscles of Kit^+/copGFP^ mice by FACS from, as previously described (Baker et al., 2016), and characterized the expression of *Chrm2* and *Chrm3* transcripts and *Tacr1* and *Tacr2* transcripts. We noted higher expression of *Chrm3* in ICC in comparison to *Chrm2* normalized to the housekeeping gene *Gapdh* (*Chrm3*: 0.043 ± 0.001; *Chrm2*: 0.029 ± 0.002, *P* = 0.001, *n* = 4). *Chrm3* transcripts were also higher in ICC relative to unsorted cells (total cell population). *Tacr1* was also highly expressed in ICC (*Tacr1*: 0.06 ± 0.01, *n* = 4), and expression of *Tacr2* was not resolved in ICC. Thus, the dominant receptor transcripts in ICC were *Chrm3* and *Tacr1*.

### Cholinergic regulation of Ca^2+^ transients in ICC-DMP

**Figure 4.**
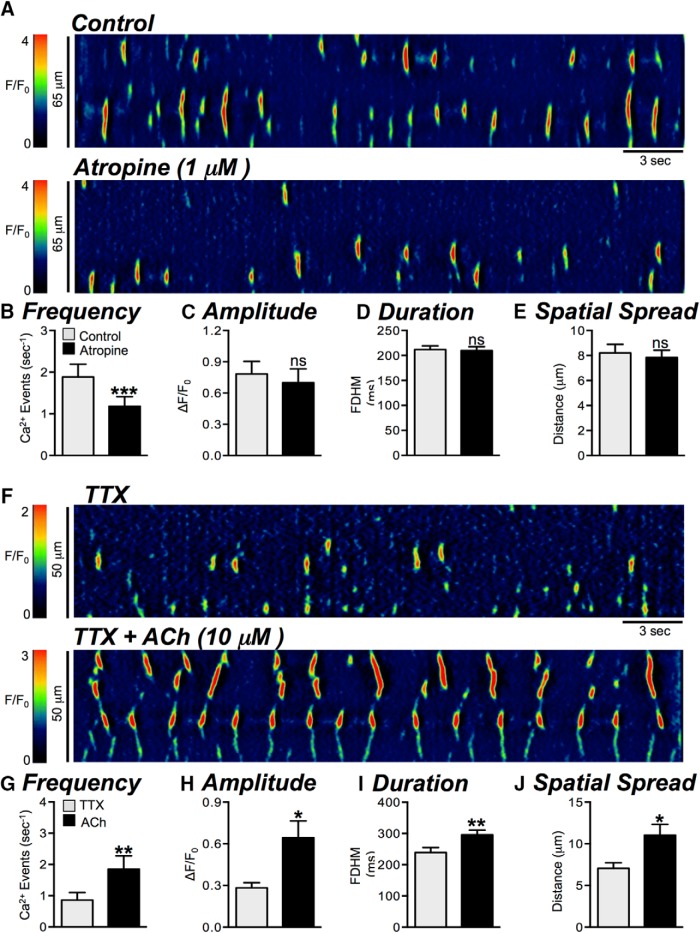
Modulation of basal Ca^2+^ transients by cholinergic input. ***A***, Representative ST maps showing the effects of atropine (1 μM) on basal Ca^2+^ transient activity in ICC-DMP. ***B–E***, Summary graphs showing the effect of atropine on the frequency (***B***), amplitude (***C***), duration (***D***), and spatial spread (***E***) of basal Ca^2+^ transients in ICC-DMP (*n* = 5, c = 13). ***F***, Representative ST maps showing the effects of ACh (10 μM; in the presence of TTX) on Ca^2+^ transients in ICC-DMP. ***G–J***, Summary graphs showing the effect of ACh (in the presence of TTX) on the frequency (***G***), amplitude (***H***), duration (***I***), and spatial spread (***J***) of Ca^2+^ transients in ICC-DMP (*n* = 5, c = 9); ns = *p* > 0.05, *p < 0.05, **p < 0.01, ***p < 0.001.

Atropine (1 μM) decreased the frequency of basal Ca^2+^ transients from 1.9 ± 0.31 to 1.2 ± 0.2 events s^−1^ (Fig. 4*A*,*B*, *p* = 0.0005, *n* = 5, c = 13). No significant effects on the other parameters of Ca^2+^ transients were noted: amplitude (*p* = 0.39), duration (*p* = 0.83) or spatial spread (*p* = 0.53; Fig. 4*B–E*, *n* = 5, c = 13). When cholinergic stimulation was initiated by exogenous acetylcholine (ACh, 10 μM; in the presence of TTX, 1 μM), Ca^2+^ transients increased markedly. ACh increased the frequency of Ca^2+^ transients from 0.85 ± 0.2 to 1.85 ± 0.4 events s^−1^ (Fig. 4*F*,*G*, *p* = 0.003, *n* = 5, c = 9), and Ca^2+^ transient amplitude increased from 0.3 ± 0.04 to 0.6 ± 0.1 ΔF/F_0_ (Fig. 4*H*, *p* = 0.042, *n* = 5, c = 9). ACh increased the duration of Ca^2+^ transients from 240 ± 16.1 to 296 ± 15.3 ms (Fig. 4*I*, *p* = 0.008, *n* = 5, c = 9). The spatial spread of Ca^2+^ transients also increased in response to ACh, sometimes leading to propagating Ca^2+^ waves in contrast to more spatially limited events. Spatial spread increased from 7.1 ± 0.7 to 11 ± 1.3 μm (Fig. 4*F*,*J*, *p* = 0.017, *n* = 5, c = 9).

### The effects of atropine on EFS-evoked excitatory Ca^2+^ response in ICC-DMP

**Figure 5.**
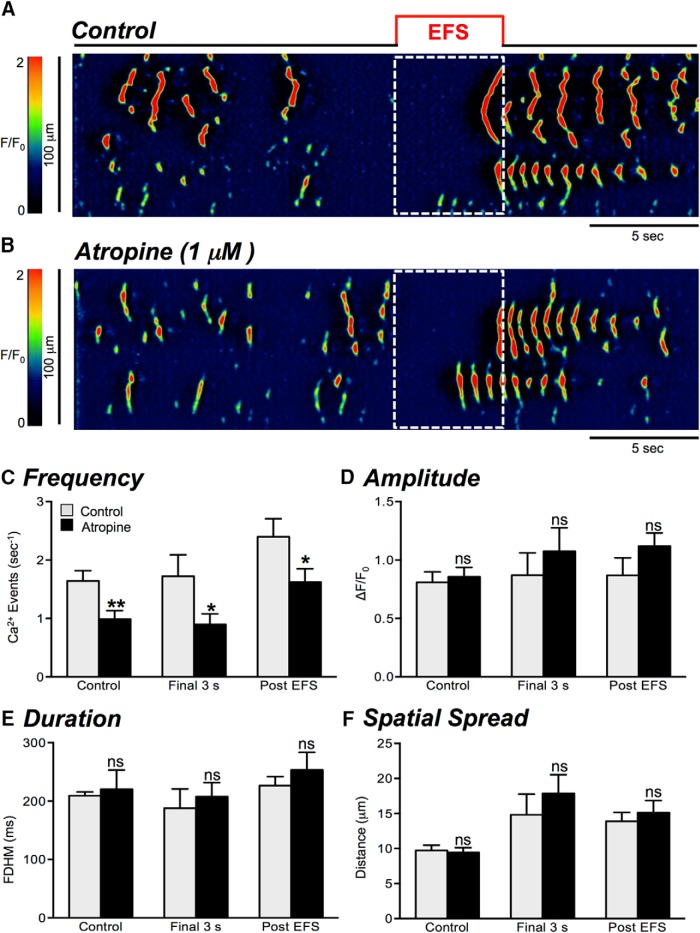
Effects of atropine on Ca^2+^ transient responses to EFS. ***A***, ***B***, Representative ST maps showing the effects of atropine (1 μM) on Ca^2+^ transients in ICC-DMP in response to nerve stimulation (EFS; 10 Hz; 5 s; indicated by the red line and dotted white box in ST maps). ***C–F***, Summary data showing the effects of atropine (1 μM) on Ca^2+^ transients during EFS: frequency (***C***), amplitude (***D***), duration (***E***), and spatial spread (***F***) in ICC-DMP during control conditions, during the excitatory period of EFS (final 3 s), and during the post-EFS period (5 s), *n* = 5, c = 21; ns = *p* > 0.05, *p < 0.05, **p < 0.01.

Next, we investigated the extent of regulation exerted by cholinergic neurotransmission on Ca^2+^ transients in ICC-DMP. EFS (10 Hz, for 5 s) in the presence of atropine (1 μM) resulted in a decrease in the frequency of Ca^2+^ transients during stimulation (final 3 s), from 1.7 ± 0.4 to 0.9 ± 0.2 events s^−1^ (Fig. 5*A–C*, *p* = 0.042, *n* = 5, c = 21). Atropine also decreased the Ca^2+^ transient frequency during the post stimulus period, from 2.4 ± 0.3 to 1.6 ± 0.2 events s^−1^ (Fig. 5*A–C*, *p* = 0.037, *n* = 5, c = 21). This suggests that cholinergic neurotransmission can also affect the poststimulus excitatory period in ICC-DMP. There were no significant changes in Ca^2+^ transient amplitude (Fig. 5*D*, *p* = 0.46, *p* = 0.19), duration (Fig. 5*E*, *p* = 0.63, *p* = 0.42), or spatial spread (Fig. 5*F*, *p* = 0.44, *p* = 0.56) during either the final 3 s of EFS or during the poststimulus period in the presence of atropine (*n* = 5, c = 21).

### Neurokinins control over basal Ca^2+^ signaling in ICC-DMP

**Figure 6.**
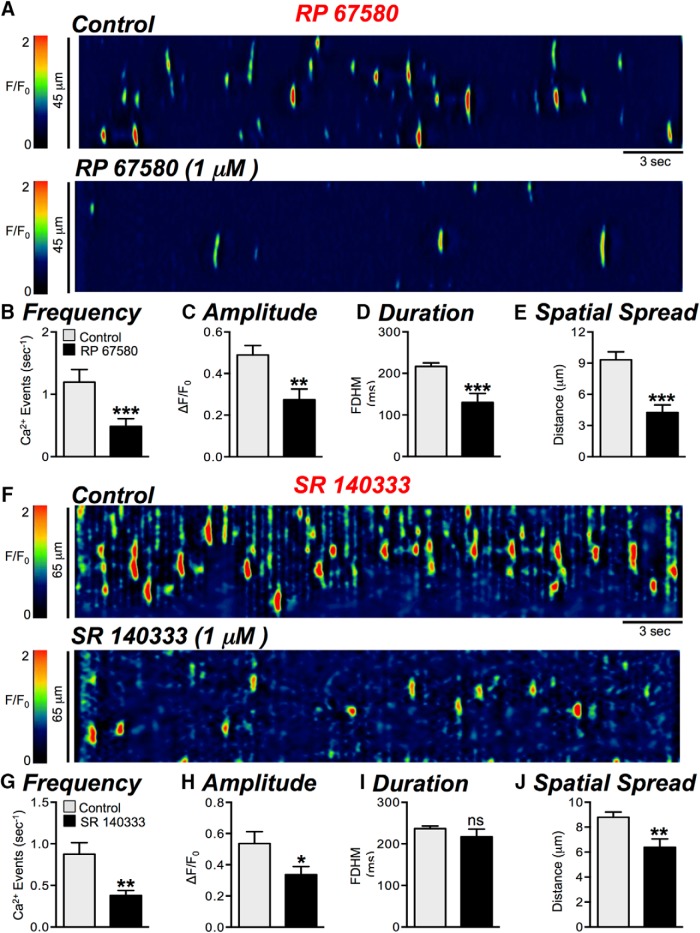
Effects of neurokinin receptor (NK1) antagonists on basal Ca^2+^ transients. ***A***, Representative ST maps showing the inhibitory effects of the NK1 receptor antagonist, RP 67580 (1 μM), on Ca^2+^ transients in ICC-DMP. ***B–E***, Summary graphs showing the effects of RP 67580 on the frequency (***B***), amplitude (***C***), duration (***D***), and spatial spread (***E***) of Ca^2+^ transients in ICC-DMP (*n* = 11, c = 27). ***F***, Representative ST maps showing the inhibitory effects of the NK1 receptor antagonist, SR 140333 (1 μM), on Ca^2+^ transients in ICC-DMP. ***G–J***, Summary graphs showing the effects of 1 μM SR 140333 on the frequency (***G***), amplitude (***H***), duration (***I***), and spatial spread (***J***) of Ca^2+^ transients in ICC-DMP (*n* = 4, c = 14); ns = *p* > 0.05, *p < 0.05, **p < 0.01, ***p < 0.001.

NK1 receptors are the major neurokinin receptors expressed in ICC, and results from this study confirmed previous reports (Sternini et al., 1995; Iino et al., 2004). Therefore, contributions of neurokinins to EFS responses in ICC-DMP were first evaluated with neurokinin 1 (NK1) receptor antagonists. RP 67580 (1 μM) dramatically reduced basal Ca^2+^ transients in ICC-DMP from 1.2 ± 0.2 to 0.5 ± 0.1 events s^−1^ (Fig. 6*A*,*B*, *p* = 0.0003, *n* = 11, c = 27). The amplitude (*p* = 0.0039), duration (*p* = 0.002), and spatial spread (*p* = 0.005) of Ca^2+^ transients were also significantly depressed by RP 67580 (Fig. 6*C–E*, n = 11, c = 27). SR 140333 (1 μM), another NK1 receptor antagonist, also inhibited basal Ca^2+^ transients in ICC-DMP, reducing frequency from 0.9 ± 0.1 to 0.4 ± 0.06 events s^−1^ (Fig. 6*F–J*, *p* = 0.006, *n* = 4, c = 14). Amplitudes (*p* = 0.042) and spatial spread (*p* = 0.003) of Ca^2+^ transients were also significantly decreased by SR 140333 (Fig. 6*H*,*J*, *n* = 4, c = 14). A selective NK2 receptor antagonist, MEN 10376 (1 μM), had no effect on Ca^2+^ transients in ICC-DMP [i.e., frequency (*p* = 0.081), amplitude (*p* = 0.67), duration (*p* = 0.24), or spatial spread (*p* = 0.21), *n* = 5, c = 9; data not shown].

**Figure 7.**
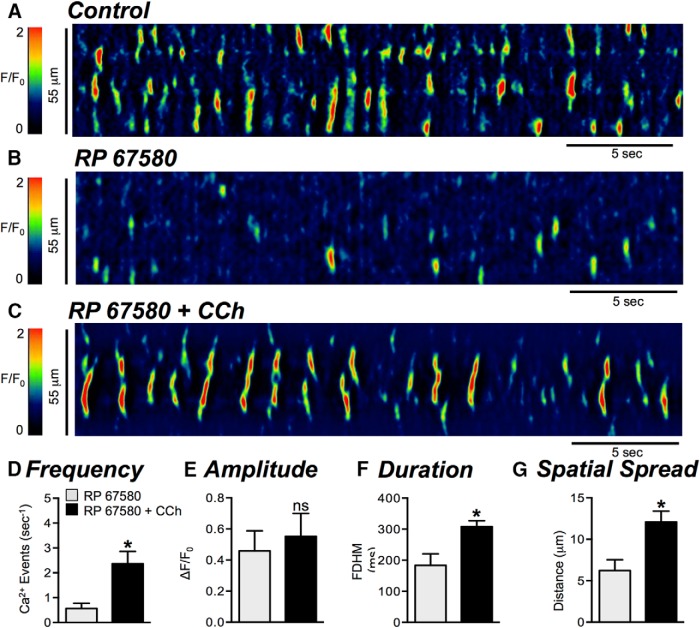
NK1 antagonist does not interfere with Ca^2+^ release mechanisms. ***A***, ***B***, Representative ST maps showing the effect of the NK1 antagonist RP 67580 (1 μM) on Ca^2+^ transients in ICC-DMP. ***C***, ST map showing that in the presence of RP 67580, CCh (10 μM) strongly activates Ca^2+^ transients. ***D–G***, Summary graphs showing the effects of CCh on Ca^2+^ transient parameters: frequency (***D***), amplitude (***E***), duration (***F***), and spatial spread (***G***) in ICC-DMP in the presence of RP 67580 (*n* = 3, c = 6); *p < 0.05.

After inhibition of Ca^2+^ transients with RP 67580 (Fig. 7*A*,*B*), carbachol (CCh; 10 μM) persisted in enhancing Ca^2+^ transient firing frequency from 0.6 ± 0.2 to 2.4 ± 0.5 events s^−1^ (Fig. 7*C*,*D*, *p* = 0.018, *n* = 3, c = 6). The duration of Ca^2+^ transients was increased by CCh from 183.4 ± 37.4 to 308.3 ± 18.9 ms (Fig. 7*F*, *p* = 0.035, *n* = 3, c = 6) and the spatial spread of Ca^2+^ transients was also increased from 6.2 ± 1.3 to 12.1 ± 1.3 μm (Fig. 7*G*, *p* = 0.016, *n* = 3, c = 6). CCh also increased Ca^2+^ transients after treatment with SR 140333 (data not shown). These results show that the effects of the NK1 antagonists were not due to off-target effects, such as inhibition of IP_3_-dependent signaling or Ca^2+^ release from intracellular stores.

**Figure 8.**
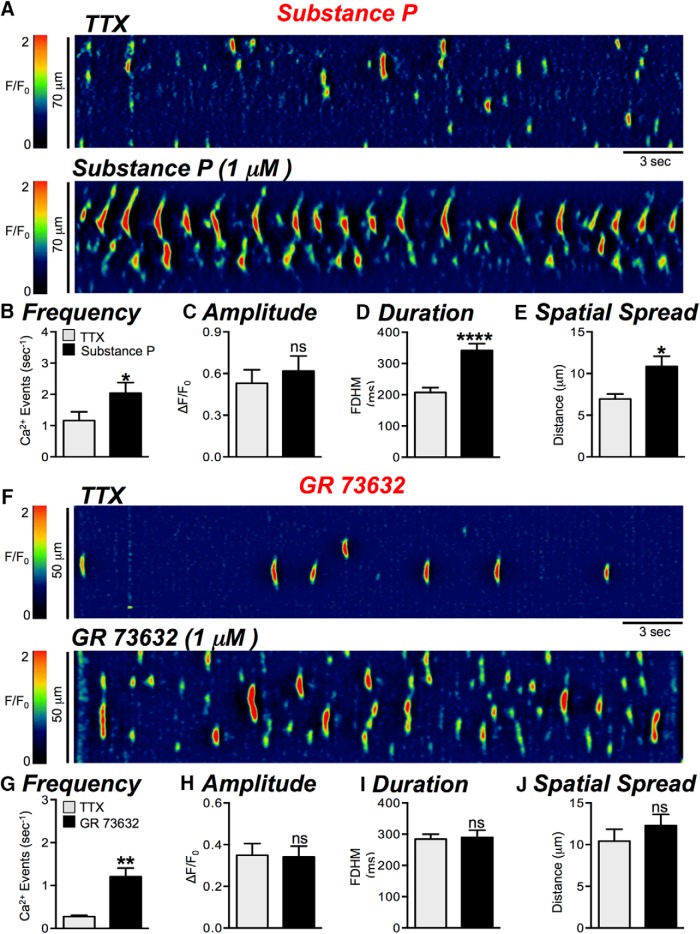
Neurokinin receptor (NK1) agonists activate Ca^2+^ transients. ***A***, Representative ST maps showing the excitatory effects of SP (1 μM; in the presence of TTX) on Ca^2+^ transients in ICC-DMP. ***B–E***, Summary graphs showing the effects of SP (in the presence of TTX) on the frequency (***B***), amplitude (***C***), duration (***D***), and spatial spread (***E***) of Ca^2+^ transients in ICC-DMP. ***F***, Representative ST maps showing the excitatory effects of the NK1 receptor agonist GR 73632 (1 μM; in the presence of TTX) on Ca^2+^ transients in ICC-DMP (*n* = 4, c = 10). ***G–J***, Summary graphs quantifying the effect of GR 73632 on the frequency (***G***), amplitude (***H***), duration (***I***), and spatial spread (***J***) of basal Ca^2+^ transient activity in ICC-DMP (*n* = 4, c = 9); ns = *p* > 0.05, *p < 0.05, **p < 0.01, ****p < 0.0001.

The observations above suggest that Ca^2+^ signaling in ICC-DMP can be modulated by neurokinins via NK1, but not NK2 receptors. Regulation by neurokinins was further tested by application of NK1 agonists. SP (1 μM, in the presence of TTX) increased Ca^2+^ transients significantly (Fig. 8*A–E*); frequency increased from 1.2 ± 0.3 to 2 ± 0.3 events s^−1^ (Fig. 8*B*, *p* = 0.042, *n* = 4, c = 10), duration increased from 207 ± 15.9 to 342 ± 21.8 ms (Fig. 8*D*, *p* < 0.0001, *n* = 4, c = 10) and spatial spread increased from 7 ± 0.6 to 10.9 ± 1.2 μm (Fig. 8*E*, *p* = 0.0199, *n* = 4, c = 10). A more selective NK1 agonist, GR 73632 (1 μM, in the presence of TTX) also increased the frequency of Ca^2+^ transients from 0.3 ± 0.03 to 1.2 ± 0.2 events s^−1^ (Fig. 8*F*,*G*, *p* = 0.0014, *n* = 4, c = 9), but effects on amplitude (*p* = 0.92), duration (*p* = 0.78), or spatial spread (*p* = 0.42) were not changed significantly (Fig. 8*H–J*, *n* = 4, c = 9). These data suggest that neurokinins are released tonically in small intestinal muscles, and responses of ICC-DMP to neurokinins are mediated largely by NK1 receptors.

### The effects of RP 67580 on Ca^2+^ responses evoked by EFS n ICC-DMP

**Figure 9.**
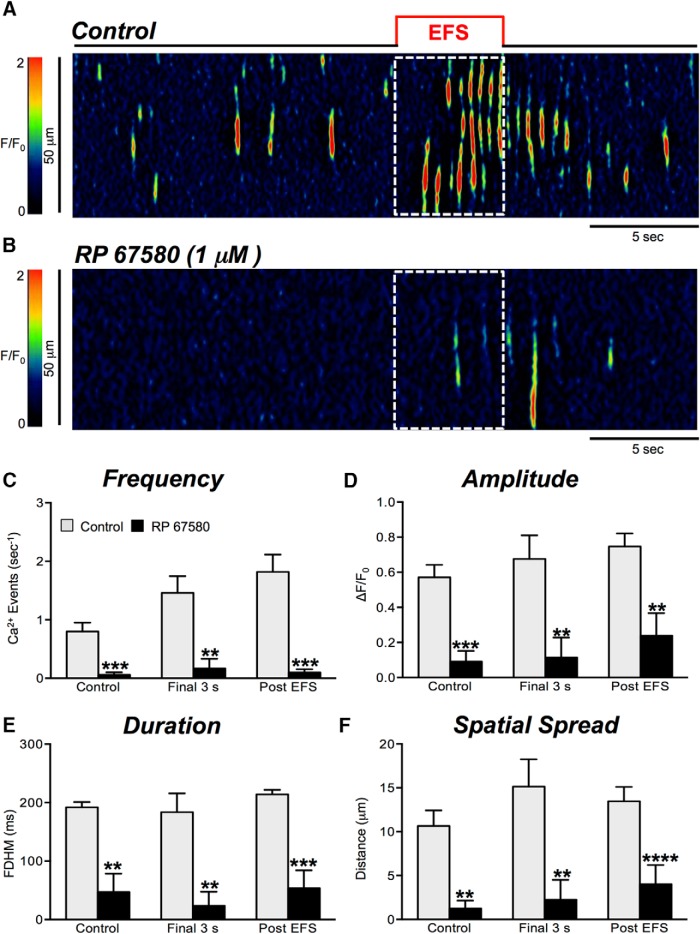
Effects of NK1 receptor antagonist on Ca^2+^ transient responses to EFS. ***A***, ***B***, Representative ST maps showing the inhibitory effects of NK1 antagonist, RP 67580 (1 μM), on Ca^2+^ transients in response to nerve stimulation (EFS at 10 Hz for 5 s; indicated by the red line and dotted white box in ST maps). ***C–F***, Summary data showing the inhibitory effects of RP 67580 (1 μM) on Ca^2+^ transient frequency (***C***), amplitude (***D***), duration (***E***), and spatial spread (***F***) in ICC-DMP during the control period, during the final 3 s of EFS, and during the post-EFS period (5 s), *n* = 4, c = 11. Note: RP 67580 reduced all Ca^2+^ transient parameters significantly; ns = *p* > 0.05, *p < 0.05, **p < 0.01.

We also tested whether NK1 receptors mediate Ca^2+^ responses in ICC-DMP evoked by EFS. RP 67580 (1 μM; Fig. 9*A*,*B*) caused a dramatic decrease in the Ca^2+^ responses to EFS (Fig. 9*C–F*). The frequency of Ca^2+^ transients during the final 3 s of EFS period was reduced from 1.5 ± 0.3 to 0.2 ± 0.17 events s^−1^ (Fig. 9*C*, *p* = 0.0015, *n* = 4, c = 11). During the post-EFS period, the frequency of Ca^2+^ transients was also significantly reduced from 1.8 ± 0.3 to 0.1 ± 0.05 events s^−1^ (Fig. 9*C*, *p* = 0.002, *n* = 4, c = 11). Ca^2+^ transient amplitude, duration and spatial spread during the final 3 s EFS and post-EFS periods were also inhibited (Fig. 9*D–F*, *n* = 4, c = 11).

**Figure 10.**
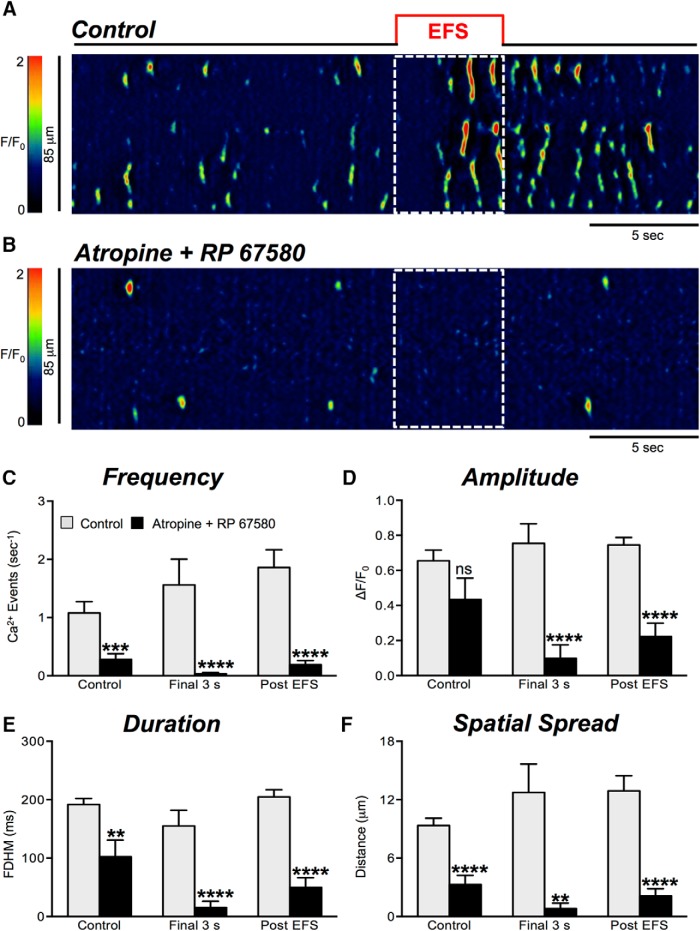
Cholinergic and NK1 receptor antagonists inhibit Ca^2+^ transients elicited by EFS in ICC-DMP. ***A***, ***B***, Representative ST maps showing the inhibitory effects of combining cholinergic and neurokinin antagonists (atropine and RP 67580; both 1 μM) on Ca^2+^ transients in ICC-DMP during EFS (10 Hz; 5 s). ***C–F***, Summary data of Ca^2+^ transient parameters showing the inhibitory effects of atropine and RP 67580 on Ca^2+^ transient frequency (***C***), amplitude (***D***), duration (***E***), and spatial spread (***F***) in ICC-DMP during the control period, during the final 3 s of EFS, and during the post-EFS period (5 s), *n* = 4, c = 20. Note: combination of atropine and RP 67580 abolished all Ca^2+^ transient parameters significantly; ns = *p* > 0.05, **p < 0.01, ***p < 0.001, ****p < 0.0001.

When cholinergic and NK1 receptors were both antagonized by adding both atropine (1 μM) and RP 67580 (1 μM), pronounced inhibition of Ca^2+^ transients persisted during the final 3 s of EFS and during the post stimulus period, as shown in Figure 10*A–F* (*n* = 4, c = 20).

### Cholinergic and neurokinin mediated excitatory responses after blocking nitrergic and purinergic transmission

**Figure 11.**
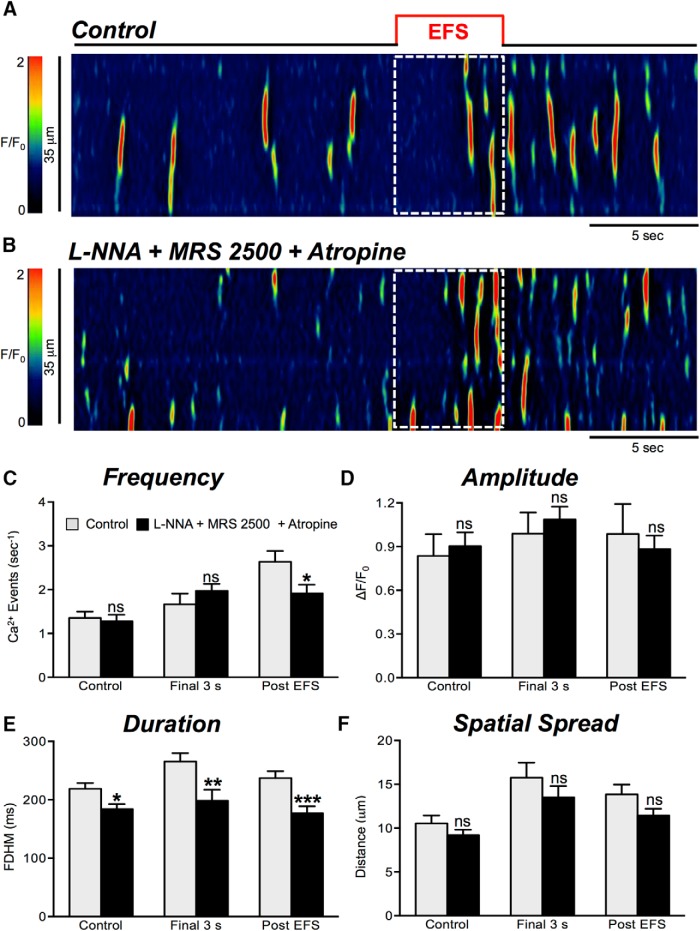
Excitatory responses are modestly reduced by atropine. ***A***, ***B***, Representative ST maps showing the inhibitory effects of atropine (1 μM) on responses to EFS (10 Hz; 5 s; indicated by the red line and dotted white box in ST maps). In this experiment L-NNA (100 μM) and the P2Y1 receptor antagonist, MRS 2500 (1 μM), were present. ***C–F***, Summary data showing the effects of a combination of L-NNA (100 μM), MRS 2500 (1 μM), and atropine (1 μM) on Ca^2+^ transient frequency (***C***), amplitude (***D***), duration (***E***), and spatial spread (***F***) in ICC-DMP during the control period, during the final 3 s of EFS, and during the post-EFS period (5 s), *n* = 7, c = 26; ns = *p* > 0.05, **p < 0.01, ***p < 0.001.

Nitrergic and purinergic antagonists N-ω-nitro-l-arginine (L-NNA, 100 μM) and MRS 2500 (1 μM) were used to examine excitatory neural regulation of Ca^2+^ transients in ICC-DMP after blocking major inhibitory pathways of neurotransmission. In the presence of L-NNA, MRS 2500, and atropine (1 μM), Ca^2+^ transient frequency (Fig. 11*C*, *p* = 0.29, *n* = 7, c = 26), amplitude (Fig. 11*D*, *p* = 0.57, *n* = 7, c = 26), and spatial spread (Fig. 11*F*, *p* = 0.3, *n* = 7, c = 26) in the final 3-s period were not significantly affected. Ca^2+^ transient duration decreased significantly from 266 ± 14.15 to 199 ± 18.9 ms (Fig. 11*E*, *p* = 0.007, *n* = 7, c = 26) in the final 3-s period. In the presence of L-NNA, MRS 2500, and atropine, Ca^2+^ transients in the poststimulus period were not reduced in amplitude (Fig. 11*D*, *p* = 0.64, *n* = 7, c = 26) or spatial spread (Fig. 11*F*, *p* = 0.088, *n* = 7, c = 26). However, the frequency of Ca^2+^ transients was reduced during the poststimulus period from 2.6 ± 0.25 to 1.9 ± 0.2 events s^−1^ (Fig. 11*C*, *p* = 0.03, *n* = 7, c = 26) and duration decreased from 237 ± 11.6 to 177 ± 11.9 ms (Fig. 11*E*, *p* = 0.0008, *n* = 7, c = 26).

**Figure 12.**
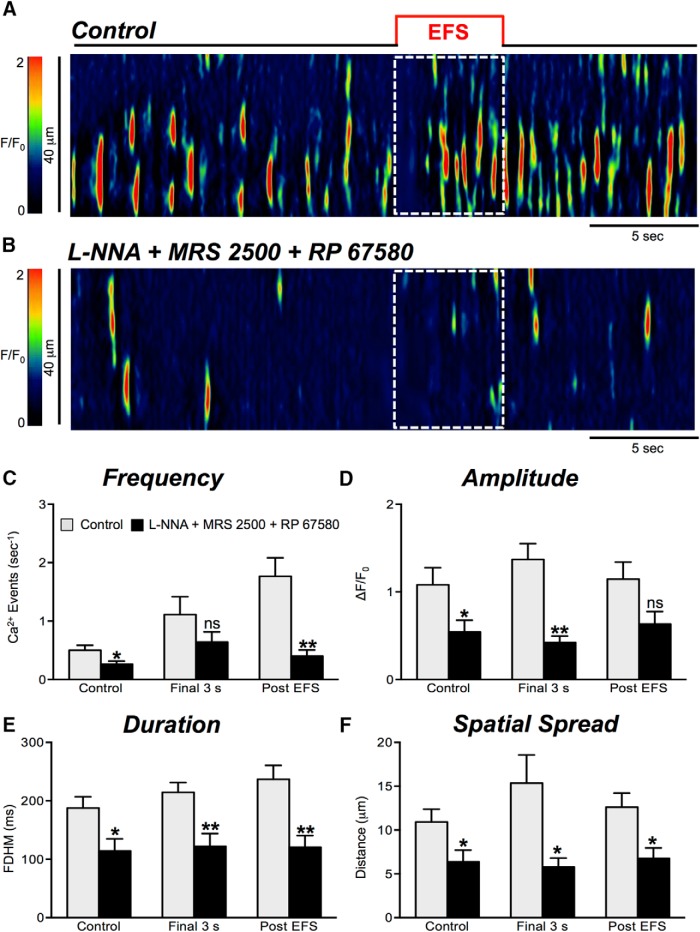
Excitatory responses are strongly attenuated by NK1 antagonist. ***A***, ***B***, Representative ST maps showing the inhibitory effects of RP 67580 (1 μM), in the presence of nitric oxide synthase inhibitor L-NNA (100 μM) and purinergic P2Y1 receptor antagonist (MRS 2500; 1 μM) on Ca^2+^ transients in response to nerve stimulation (EFS at 10 Hz 5 s; indicated by the red line and dotted white box in ST maps). ***C–F***, Summary data showing the effects of a combination of L-NNA, MRS 2500, and RP 67580 on Ca^2+^ transient frequency (***C***), amplitude (***D***), duration (***E***), and spatial spread (***F***) in ICC-DMP during the control period, during the final 3 s of EFS, and during the post-EFS period (5 s), *n* = 4, c = 13; ns = *p* > 0.05, **p < 0.01.

We next examined the neurokinin input into EFS-mediated excitatory responses in ICC-DMP in the presence of blockers of nitrergic and purinergic neurotransmission. With L-NNA, MRS 2500, and RP 67580 present, responses to EFS were significantly reduced in amplitude, duration, and spatial spread of Ca^2+^ transients during the final 3 s of EFS (Fig. 12*D–F*, *n* = 4, c = 13). The amplitude decreased from 1.4 ± 0.2 to 0.42 ± 0.8 ΔF/F_0_ (*p* = 0.002; Fig. 12*D*, *n* = 4, c = 13), the duration decreased from 214.4 ± 16.9 to 122 ± 22 ms (*p* = 0.004; Fig. 12*E*, *n* = 4, c = 13), and the spatial spread decreased from 15.4 ± 3.2 to 5.8 ± 1 μm (*p* = 0.029; Fig. 12*F*, *n* = 4, c = 13). Overall the frequency of Ca^2+^ transients in the final 3 s of the EFS period was not significantly affected (Fig. 12*A–C*, *p* = 0.21, *n* = 4, c = 13). In the presence of L-NNA, MRS 2500, and RP 67580, the frequency of Ca^2+^ transients in the post-EFS period was significantly reduced from 1.8 ± 0.3 to 0.4 ± 0.1 events s^−1^ (Fig. 12*C*, *p* = 0.006, *n* = 4, c = 13). The amplitude of Ca^2+^ transients during this period was not significantly affected (Fig. 12*D*, *p* = 0.059, *n* = 4, c = 13), but the duration of Ca^2+^ transients was reduced from 237 ± 23.9 to 121 ± 19.7 ms (Fig. 12*E*, *p* = 0.0029, *n* = 4, c = 13), and spatial spread decreased from 12.61 ± 1.6 to 6.8 ±1.2 μm (Fig. 12*F*, *p* = 0.014, *n* = 4, c = 13).

**Figure 13.**
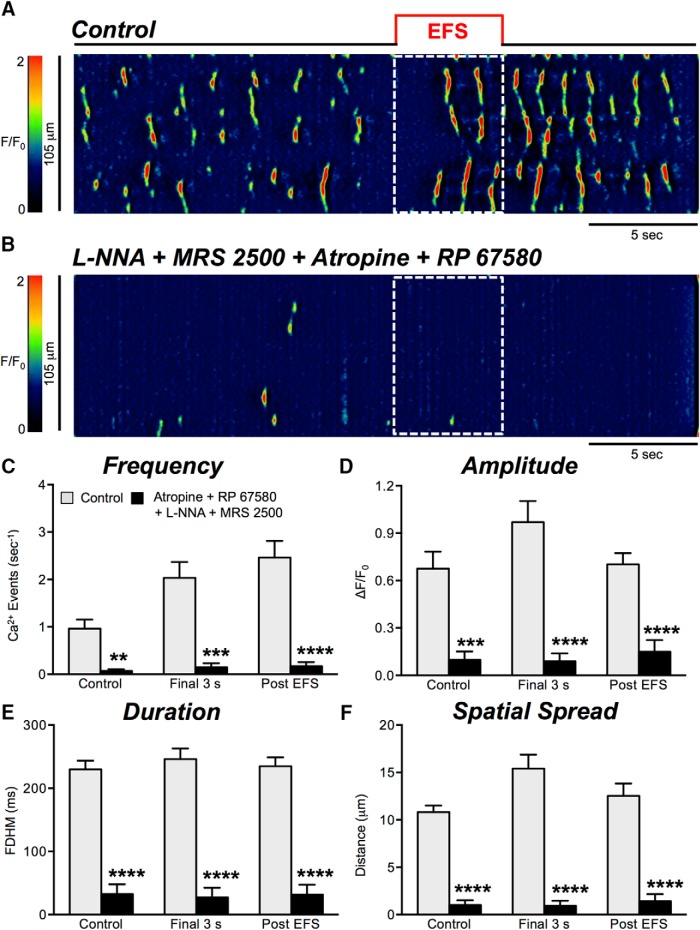
Excitatory responses to EFS are abolished by atropine and NK1 receptor antagonist. ***A***, ***B***, Representative ST maps showing inhibition of Ca^2+^ transients by atropine (1 μM) and RP 67580 (1 μM). These experiments were conducted in the presence of L-NNA (100 μM) in the presence of MRS 2500 (1 μM) during and post nerve stimulation periods (EFS at 10 Hz 5 s; indicated by the red line and dotted white box in ST maps). ***C–F***, Summary data showing the effects of a combination of L-NNA, MRS 2500, and atropine and RP 67580 on Ca^2+^ transient frequency (***C***), amplitude (***D***), duration (***E***), and spatial spread (***F***) in ICC-DMP during control conditions, excitatory periods during EFS (final 3 s), and in the post-EFS period, *n* = 5, c = 32; ns = *p* > 0.05, **p < 0.01, ***p < 0.001.

Next, we inhibited cholinergic and neurokinin transmission with atropine and RP 67580 in the presence of L-NNA and MRS 2500. Under these conditions all Ca^2+^ transients were significantly diminished across all parameters tabulated, as shown in Figure 13*A–F* (*n* = 5, c = 32).

## Discussion

Innervation of GI muscles by enteric motor nerves and the integrated firing of these neurons is essential for generating archetypal motility patterns (Spencer et al., 2016). ICC are innervated by enteric motor neurons, and their responses to neurotransmitters contribute to complex postjunctional responses of the SIP syncytium (Ward et al., 2000; Iino et al., 2004). In the case of the small intestine, ICC-DMP are an intramuscular type of ICC that are closely associated with and innervated by motor neurons (Rumessen et al., 1992; Zhou and Komuro, 1992; Wang et al., 2003b; Iino et al., 2004; Ward et al., 2006). We recently described the properties of spontaneous Ca^2+^ transients that occur in the absence of extrinsic stimuli in these cells (Baker et al., 2016). In the present study we investigated the effects of excitatory enteric motor neurotransmission on Ca^2+^ transients in ICC-DMP, because these events mediate activation of CaCC, the ion channels responsible for the electrophysiological postjunctional excitatory responses to nerve stimulation in small intestinal muscles (Zhu et al., 2011). EFS of intrinsic neurons resulted in three-component effects on Ca^2+^ transients: a brief inhibitory period (∼2 s), a period of escape from inhibition during sustained EFS, and a period of strong excitation after cessation of the stimuli (poststimulus or “rebound” excitation). The complexity of these responses is likely due to the fact that the enteric nervous system contains both inhibitory and excitatory motor neurons (Furness, 2012), and EFS can be expected to activate both classes of neurons.

In the mouse small intestine, the neurokinin component of the excitatory neural inputs to ICC-DMP was dominant. Our experiments also suggest that tonic release of neurokinins and binding to NK1 receptors is responsible for significant drive in generating the Ca^2+^ transients observed under basal conditions in ICC-DMP (Baker et al., 2016). Thus, the Ca^2+^ transients observed in the absence of applied stimuli are not “spontaneous” and do not appear to be driven intrinsically within ICC-DMP. Excitatory neurotransmitters greatly increased Ca^2+^ transients in ICC-DMP, and this mechanism likely underlies a portion of the postjunctional electrophysiological response to excitatory neural regulation (Zhu et al., 2011, 2015).

ICC-DMP are plentiful and in close contact with varicosities of enteric motor neurons in the DMP region of the small intestine (Rumessen et al., 1992; Zhou and Komuro, 1992). We confirmed that ICC express receptors required for excitatory motor neurotransmission (e.g., muscarinic and neurokinin receptors), and transcripts for M3 (*Chrm3*) and NK1 (*Tacr1*) were enriched in ICC-DMP versus unsorted cells. However, transcripts of *Chrm2* were also present, suggesting these receptors and coupling to effectors via Gi/Go may also have a role in transduction or modulation of excitatory neurotransmission. Our findings are consistent with previous studies showing muscarinic receptors and NK1 receptor expression in ICC with immunohistochemical techniques (Sternini et al., 1995; Vannucchi et al., 1997; Stadelmann et al., 1998; Iino et al., 2004; Iino and Nojyo, 2006; Ward et al., 2006; Sanders et al., 2014b).

This study demonstrates that ICC-DMP receive and transduce excitatory neural inputs in the small bowel. Previous studies predicted this finding from morphologic observations (Rumessen et al., 1992; Zhou and Komuro, 1992; Wang et al., 2003a; Iino et al., 2004; Faussone-Pellegrini, 2006; Shimizu et al., 2008) and by showing that cholinergic excitatory neural responses develop in phase with the development of ICC-DMP and blocking Kit receptors causes parallel loss of ICC and cholinergic neural responses (Ward et al., 2006). Excitatory neurotransmission caused PKCɛ translocation in ICC-DMP that was blocked by atropine (Wang et al., 2003b), demonstrating functional cholinergic innervation and muscarinic responses in these cells. ACh binding to M3 receptors can enhance Ca^2+^ release in ICC-DMP via generation of inositol 1,4,5-trisphosphate (IP_3_) which activates Ca^2+^ release from the endoplasmic reticulum (ER). All of the molecular components of this pathway are expressed in ICC, as shown by transcriptome analyses (Chen et al., 2007; Lee et al., 2017). Previous direct observation of ICC-DMP *in situ* has shown that Ca^2+^ transients are due to Ca^2+^ release from intracellular stores (e.g., ER), mediated, in part, by IP_3_R (Baker et al., 2016). Increasing Ca^2+^ release in ICC leads to activation of CaCC, and the inward current generated by thousands of ICC-DMP in whole muscles would provide a net depolarizing influence that would summate with slow wave depolarizations, increase the likelihood of action potentials being generated during the plateau phase of slow waves (i.e., period of peak depolarization), and enhance the amplitude of phasic contractions (Zhu et al., 2011).

While our observations suggest innervation and contributions from cholinergic nerves to postjunctional excitatory responses, our data also suggest that neurokinins are the dominant excitatory neurotransmitters affecting Ca^2+^ transients in ICC-DMP in the mouse small intestine. ICC-DMP are closely associated with SP containing nerve fibers, and ICC-DMP express NK1 receptors (Iino et al., 2004; Faussone-Pellegrini, 2006; Shimizu et al., 2008) which is consistent with our observation that excitatory transmission to ICC-DMP was mediated through NK1 receptors. Previous studies have shown that exposure of small intestinal muscles to SP or stimulation of motor neurons causes internalization of NK1 receptors in ICC (Lavin et al., 1998; Iino et al., 2004). Our experiments showed that two NK1 receptor antagonists greatly attenuated basal Ca^2+^ transients and suppressed responses of ICC-DMP to EFS. The strong inhibitory effects of NK1 antagonists on Ca^2+^ transients could possibly be due to off-target effects on Ca^2+^ stores or Ca^2+^ release mechanisms; however, nonspecific effects do not appear to be significant because responses to CCh on Ca^2+^ transients were intact in the presence of the NK1 antagonist, RP 67580. Taken together these findings support the importance of neurokinin signaling in shaping motility patterns in the small intestine.

The degree to which basal Ca^2+^ transients were affected by NK1 antagonists in the present study was somewhat surprising. These results suggest ongoing release of neurokinins (i.e., tonic excitation), similar in concept to the tonic inhibition phenomena observed in many GI muscles (Wood, 1972; Lyster et al., 1995). Although this phenomenon has not been described previously in the small intestine, tonic activation of NK1 receptors has been proposed in other systems (Henry et al., 1999; Jasmin et al., 2002). In the present study attenuation of Ca^2+^ transients by the NK1 receptor antagonists may be caused by continuous release of neurokinins or persistence of the ligand in the spaces between motor nerve varicosities and ICC-DMP.

The enhanced relative reliance on neurokinins for excitatory effects may be due, in part, to the high expression of NK1 receptors by ICC-DMP which does not appear to be true for intramuscular ICC in the colon (Lee et al., 2017). NK1 receptors also couple to cellular responses through activation of phospholipase C and generation of IP_3_ (Steinhoff et al., 2014). Thus, there is a signaling pathway available for the enhancement of Ca^2+^ transients in ICC-DMP. However, it should also be noted that transfection of neurokinin receptors in model cells has also been associated with activation of adenylate cyclase and production of cAMP (Steinhoff et al., 2014), a pathway not typically linked to enhanced release of Ca^2+^. Generation of cAMP and stimulation of cAMP-dependent protein kinase is known to enhance Ca^2+^ sequestration into stores by phosphorylation of phospholamban (highly expressed in ICC; Lee et al., 2017) and stimulation of SERCA (Stammers et al., 2015). Perhaps increased loading of Ca^2+^ stores contributes to augmentation of Ca^2+^ transient amplitude and spatial spread by neurokinins, and enhancing the rate of recovery of Ca^2+^ into stores after a release event, reducing the time required for a given store to generate another Ca^2+^ transient.

In summary, this study supports the idea that significant neural regulation occurs in the intramuscular class of ICC in the small intestine (ICC-DMP). As discussed above, much of the excitatory response was mediated through NK1 receptors that are expressed largely by ICC-DMP (Sternini et al., 1995; Vannucchi et al., 1997; Iino et al., 2004). Responses to EFS were attenuated by NK1 antagonists. Previous studies have shown that electrophysiological responses in ICC-DMP are linked to Ca^2+^ release events (Zhu et al., 2011; Zhu et al., 2015), suggesting that Ca^2+^ transients in ICC-DMP couple to generation of inward currents and depolarizing effects on the SIP syncytium. NK2 receptors, expressed largely by SMCs (Cipriani et al., 2011), were apparently not involved in responses of ICC-DMP to neurokinins released from nerve terminals, because an NK2 antagonist had no effect on responses. The effectiveness of neurokinins as neurotransmitters in the tunica muscularis of the small intestine may be spatially confined by concentrations achieved in postjunctional spaces to a subset of neurokinin receptors expressed by ICC-DMP.

## Synthesis

Reviewing Editor: Vivian Budnik, University of Massachusetts Decisions are customarily a result of the Reviewing Editor and the peer reviewers coming together and discussing their recommendations until a consensus is reached. When revisions are invited, a fact-based synthesis statement explaining their decision and outlining what is needed to prepare a revision will be listed below. The following reviewer(s) agreed to reveal their identity: Nick Spencer

the current manuscript addresses all the criticisms raised by reviewers.
